# Preliminary studies developing methods for the control of *Chrysomya putoria*, the African latrine fly, in pit latrines in The Gambia

**DOI:** 10.1111/tmi.12033

**Published:** 2012-11-30

**Authors:** T C Lindsay, M Jawara, U D'Alessandro, M Pinder, S W Lindsay

**Affiliations:** 1 London School of Hygiene and Tropical MedicineLondon, UK; 2 Medical Research Council UnitFajara, The Gambia; 3 Institute of Tropical MedicineAntwerp, Belgium; 4 School of Biological and Biomedical Sciences, Durham UniversityDurham, UK

**Keywords:** *Chrysomya putoria*, African latrine fly, sanitation, fly control, pit latrines, diarrhoeal diseases

## Abstract

**Objective:**

To explore ways of controlling *Chrysomya putoria*, the African latrine fly, in pit latrines. As pit latrines are a major source of these flies, eliminating these important breeding sites is likely to reduce village fly populations, and may reduce the spread of diarrhoeal pathogens.

**Methods:**

We treated 24 latrines in a Gambian village: six each with (i) pyriproxyfen, an insect juvenile hormone mimic formulated as Sumilarv® 0.5G, a 0.5% pyriproxyfen granule, (ii) expanded polystyrene beads (EPB), (iii) local soap or (iv) no treatment as controls. Flies were collected using exit traps placed over the drop holes, weekly for five weeks. In a separate study, we tested whether latrines also function as efficient flytraps using the faecal odours as attractants. We constructed six pit latrines each with a built-in flytrap and tested their catching efficiency compared to six fish-baited box traps positioned 10 m from the latrine. Focus group discussions conducted afterwards assessed the acceptability of the flytrap latrines.

**Results:**

Numbers of emerging *C. putoria* were reduced by 96.0% (95% CIs: 94.5–97.2%) 4–5 weeks after treatment with pyriproxyfen; by 64.2% (95% CIs: 51.8–73.5%) after treatment with local soap; by 41.3% (95% CIs = 24.0–54.7%) after treatment with EPB 3–5 weeks after treatment. Flytraps placed on latrines collected *C. putoria* and were deemed acceptable to local communities.

**Conclusions:**

Sumilarv 0.5G shows promise as a chemical control agent, whilst odour-baited latrine traps may prove a useful method of non-chemical fly control. Both methods warrant further development to reduce fly production from pit latrines. A combination of interventions may prove effective for the control of latrine flies and the diseases they transmit.

## Introduction

Pit latrines are common throughout much of sub-Saharan Africa (UNICEF/WHO 2012). Whilst they are preferable to open defaecation, latrines can produce prodigious numbers of flies, particularly *Chrysomya putoria*, the African latrine blowfly (Laurence 1988; Emerson *et al*. 2005). In The Gambia, an average pit latrine produces over 100,000 *C. putoria* annually (Emerson *et al*. 2005), many of which are contaminated with enterovirulent pathogens (Lindsay *et al*. 2012). As latrines are a major source of the flies, control efforts to reduce fly numbers should be targeted at latrines, which are easy to locate.

Whilst there are many ways of controlling flies (Rozendaal 1997), we know of only one design specifically for controlling *C. putoria*: the ventilated improved pit (VIP) latrine (Morgan 1977). This latrine has a small building constructed over a pit latrine with a tall ventilation pipe releasing odours from the pit. The pipe has netting screening at the top to prevent flies entering the pipe, and it captures any flies produced in the pit as they are attracted to the light at the end of the pipe. For the latrines to work, the netting over the vent pipe must remain intact. Unfortunately, this is not always the case, and in Botswana, Ghana and Tanzania, few pipes had effective screens (Curtis & Hawkins 1984; Dumpert *et al*. 2009). Moreover, the interior of the structure should be dark so that flies in the pit are attracted to the light from the vent pipe. In Zimbabwe, this was achieved by constructing the surface structure with a spiral ground plan, but in other places the superstructure has a rectangular ground plan with a door in one side, and these cannot be kept dark as people leave the doors open (Dumpert *et al*. 2009). Thus, many VIP latrines do not control flies.

In this study, we tried two approaches to fly control: latrine treatment and odour-baited traps. For latrine treatment, we tested pyriproxyfen, expanded polystyrene beads (EPB) and local soap. Pyriproxyfen is an insect growth regulator, recommended for fly control by the World Health Organization (WHO 2006). Its primary effect is to prevent metamorphosis of pupae into adults, although it also has embryogenic and reproductive effects (Invest & Lucas 2008). It is effective against the housefly *Musca domestica* (Hatakoshi *et al*. 1987; Kawada *et al*. 1987; Bull & Meola 1994; Geden & Devine 2012) and against the stable fly *Stomoxys calcitrans* (Bull & Meola 1994), so we thought it likely to be effective against *C. putoria*. We also sought to test interventions that could be applied more readily by local communities, such as expanded polystyrene beads (EPB) and local soap. Applying a layer of EPB over the faeces can markedly reduce the emergence of the mosquito *Culex quinquefasciatus* from latrines (Curtis & Minjas 1985; Reiter 1985; Curtis 2005). The layer prevents gravid females from ovipositing, and mature larvae and pupae are unable to breach the water surface and suffocate. A similar approach might be effective at reducing fly numbers. We observed that where waste water from bathing was poured down the latrine drop hole, fewer flies were produced than where this was not carried out and hypothesised that the local soap was killing fly larvae.

Our previous fieldwork demonstrated that *C. putoria* was attracted strongly to human faeces (Lindsay *et al*. 2012) and that they could be trapped using a simple plastic box trap (Lindsay *et al*. in press). We therefore tried a proof-of-principle experiment to test whether we could use the odours generated from a latrine to attract flies and trap them there. This is the first occasion we are aware of where scientists have attempted to turn a latrine into a flytrap. The acceptability of this intervention was assessed by carrying out focus group discussions (FGDs) with latrine users. This series of experiments is a pilot study to assess potential interventions for controlling *C. putoria*.

## Methods

### Study sites

Studies were carried out in the Upper River Region of The Gambia between June 2011 and February 2012. This area has a rainy season from June to October followed by a long dry season. It is an area of open Sudanian savannah where most people live in villages in houses of mud or cement walls and thatched or metal roofs. Toilets are usually pit latrines, although open defaecation does occur. Latrine treatments were carried out in Dampha Kunda village, and the other studies were carried out in Kundam Demba village.

### Treatment interventions

Consent by latrine owners was sought and, after approval, exit traps based on a design by Muirhead-Thomson (Muirhead Thomson 1948; Service 1976) were left over the drop holes of latrines between 09:00 and 16:00 h. These traps consisted of a steel-rod framed cube (40 **×** 40 **×** 40 cm) covered in cotton-mosquito netting. The funnelled entrance on the bottom face of the trap had netting flaps (40 **×** 10 cm) extending outwards from the base to prevent flies crawling out of the trap if the surface of the pit latrine was uneven. On collection, the entrance hole of each trap was plugged to prevent any flies escaping.

Latrine pits were 2.5–7.0 m deep and 1.0–1.6 m in diameter. The slabs placed over the pit were 2.0 m^2^ in area and 0.15 m thick and made from concrete reinforced with either 1-cm-diameter iron rods or 15-cm-diameter wooden logs. Drop holes were 15 cm in diameter. Only latrines producing >10 adult *Chrysomya* spp. on one day were selected for further study. 24 latrines were randomised into four groups: six were treated with 500 g Sumilarv 0.5G (formulated as a 0.5% w/w granule, Sumitomo Chemical Co., Osaka, Japan); 6 with 15 l of 2-mm-diameter EPB (Custompac, Castleford, UK); and six with two 100-g local soap balls each containing 20 g of caustic soda, 60-g groundnut oil and 20-g maize corn husk ash per ball. Six latrines were left untreated as controls. Each Sumilarv 500-g sachet was mixed with 1 l of water. Soap balls were completely dissolved in 3 l of boiling water and allowed to cool before application. Treatments were added to the latrines by putting a hand down the drop hole and pouring the treatment over the excrement surface using an empty tomato-paste tin as a container. Each latrine was treated once, and an exit trap placed over the drop hole between 08:00 and 16:45 h to collect flies once a week for 5 weeks.

### Flytrap intervention

**Figure 1:**
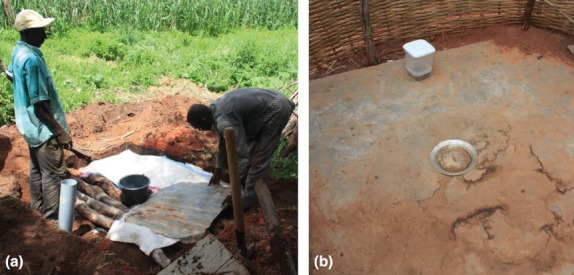
Construction of a latrine flytrap. (a) Here, a membrane is applied over a row of logs before concreting. In the centre is the bucket acting as a mould for the drop hole and to the left is the vent pipe on which the trap is positioned. (b) The completed latrine with drop hole cover and flytrap in place.

**Figure 2:**
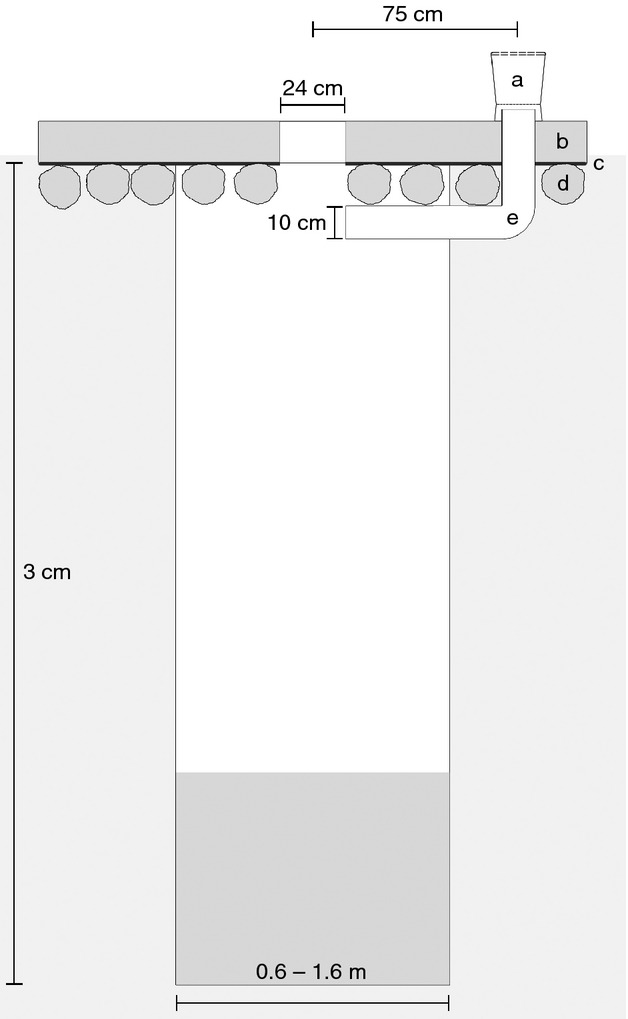
Cross-section of the finished latrine with flytrap in place over the vent pipe. (a) the flytrap, (b) 20-cm-deep concrete slab, (c) single layer of nylon rice bags acting as membrane, (d) locally found logs roughly 15 cm in diameter, (e) 10-cm-diameter grey plastic vent pipe.

To prove that pit latrines could be adapted to collect flies, six pit latrines were built in the traditional fashion in Kundam Demba (Figures 1 and 2). Circular pits were dug 3 m deep and 0.6 m in diameter. A 1.6 **×** 1.6 **×** 0.2 m shallow tray for the logs was dug in the sandy soil around the top of the pit. Wooden logs, approximately 1.5 m long and 0.15 m in diameter, were placed in parallel in the tray to act as the reinforcing structure and covered with old nylon rice bags. A bucket, 24 cm diameter, was placed directly over the centre of the pit to be used as the drop hole mould. A 10-cm-diameter grey L-bend pipe vented air from the latrine and exited into the slab (Figure 2). Four wooden planks were placed around the sides of the tray, and wet concrete was poured into the mould. The concrete mixture consisted of two 36-kg bags of cement, 0.6 m^3^ of sharp sand, 0.6 m^3^ of assorted sizes of gravel and approximately 40 l of water and was allowed to set overnight. If the latrine area was not already protected, a 2-m-high superstructure made from local wooden fencing (*krinting*) was built around the slab. The bucket and wooden planks were removed, and the latrine was ready to use. After 1–2 weeks, the walls of the pit collapsed gradually under their own weight. Latrine owners were each given a 0.24-m-diameter lid (a local tea tray) as a cover for the drop hole. Each latrine was used by an average of six people for 15 weeks before flytraps were attached to the pit latrines' vent pipes for 12 consecutive days.

Both the latrine odour-baited flytrap and the fish odour-baited flytrap were based on the same box trap (Lindsay *et al*. 2012); a semi-transparent polypropylene 3-l box (17 cm^3^) with a snap-top white opaque lid (Whitefurze, Coventry, UK) perforated with ten 1.6-cm-diameter entrance holes. Each hole had a white paper conical collar attached with a diameter of 0.6 cm protruding into the trap. The latrine flytrap had a 10 **×** 10 cm square removed from its base, which was covered with cotton netting and fitted over the vent pipe.

The fish-baited trap was baited with 50 g of raw catfish, shown to be attractive to *C. putoria* in earlier studies (Lindsay *et al*. 2012). The fish was placed in a 250 cm^3^ white plastic pot (6 cm high, 9 cm diameter; W. K. Thomas, Chessington, UK), covered with a cotton-netting lid, secured by an elastic band and placed on the floor of the trap. To find a location for the fish-baited trap, a bottle was spun next to each latrine and a standard flytrap baited with fish positioned 10 m away from the latrine in the direction indicated by the mouth of the bottle. Traps were set at 09:00 h and collected at 17:00 h on the same day. Flies were killed by freezing at −20 °C for 30 min, identified to species, sexed and counted.

### Focus group discussions

FGDs (Dawson *et al*. 1993) were conducted one week after the flytraps were installed. The owners and users of the six latrines were invited to discuss the latrines. Questions centred around the topics of (i) general feedback regarding important concerns of the user, (ii) observed changes in fly numbers, (iii) use and maintenance of the trap and (iv) a discussion on what they felt needed to be changed to meet their needs in future designs.

Discussions lasted 30–45 min and were held in Fula, the main language in the village. The three groups were as follows: (i) three to six men (aged 19–62 years old), (ii) three to six women (aged 25–50 years old) and (iii) ten children (aged 10–16 years old). The children had a larger group size to recreate a more familiar classroom environment. Each discussion always had only one representative of each latrine. Children received questions tailored to their age although the topics discussed remained the same.

A trained moderator asked a series of set questions and helped guide the discussion; a supervising moderator who sat outside the circle of participants with a translator and wrote notes as the translator described the discussion. The supervising moderator interjected only if the moderator skipped a question or he wanted to explore a particular subject further than prescribed in the question line. Discussions were recorded using a cassette recorder and transcribed by a transliterator. The translator translated the transcript into English, and both the transcript and translation were reviewed by the moderator and supervising moderator for mistakes or inaccuracies.

### Statistical analyses

It is unlikely that any of the interventions we tested were primarily killing adult flies. For this reason, any effective intervention would be expected to reduce the adult fly population a few weeks after treatment. Thus, total fly counts were compared for 0–2 weeks with 3–5 weeks after treatment, in comparison with fly counts from untreated latrines, using chi-square statistics. General estimating equations were used to account for repeated measures, and a negative binomial model used with a log link function to compute mean values for flies/catch. Statistical analysis used SPSS version 19.0.

### Ethical procedures

Ethical approval for this study was provided by the Joint Gambian Government and Medical Research Council's Laboratories in The Gambia Ethics Committee as well as the London School of Hygiene and Tropical Medicine's Ethics Committee.

## Results

### Latrine interventions

Of the 2050 insects collected from latrines, 96.83% were *C. putoria* (*n* = 1985), 2.68% were *Musca domestica* (*n* = 55), 0.39% were *Musca sorbens* (*n* = 8) and 0.10% (*n* = 2) were *Sarcophaga* spp.

**Figure 3:**
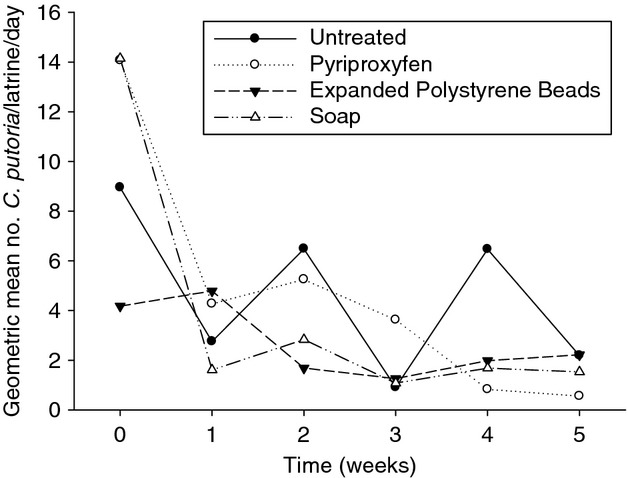
Impact of interventions on the number of adult *C. putoria* collected from latrines.

The number of *C. putoria* collected from latrines treated with pyriproxyfen declined to a plateau from week 1–3 before declining to a lower threshold in weeks 4 and 5 (Figure 3). With local soap or EPB, fly numbers declined to a threshold after about three weeks. Comparing the difference in fly numbers between the first three collections (weeks 0–2) and the last three (weeks 3–5) of the treatment compared to the control, pyriproxyfen was the most effective at reducing fly numbers (odds ratio, OR = 0.111, 95% confidence intervals, CIs = 0.086–0.144; χ^2^ = 309.48, *P* < 0.001), followed by soap (OR = 0.358, 95% CIs = 0.265–0.482, χ^2^ = 45.87, *P* < 0.001) and EPB (OR = 0.587, 95% CIs = 0.453–0.760; χ^2^ = 15.95, *P* < 0.001). The protective efficacy of pyriproxyfen was even more pronounced when the last two weeks of collection were compared with the first 4 weeks (OR = 0.040, 95% CIs = 0.028–0.055; χ^2^ = 516.52, *P* < 0.001).

### Flytrap intervention

The estimated mean number of *C. putoria* collected from latrines was 4.02 flies/catch (95% CIs = 2.66–5.38 flies/catch) compared with 8.52 flies/catch (95% CIs = 5.81–11.23 flies/catch) from fish-baited traps (Wald = 5.020, *P* = 0.025).

### Focus group discussions

Five FGDs were conducted, two with women, two with men and one with children. Most latrine users agreed there were fewer flies due to the flytrap and less malodorous odours due to the drop hole lid. Men thought that the vent pipe redirected the air away from where the user was standing and made the initial blast of hot air when opening the drop hole lid more bearable. Women described the smell from the latrines as a cause of ill health.

Importantly, children under 5 years old were not allowed by their mothers to use the latrine for fear of them falling down the drop hole or dirtying the latrine ‘especially if they have diarrhoea’. These children were expected to defaecate outside the house, and the mother to then tidy up the mess and deposit it in the latrine. One participant of the men's group mentioned that even children over 5 years old would often go into the bush to defaecate instead of the latrine.

All groups were asked what they would do if the traps broke. The children and women's groups both said they would look to the men to sort the matter. The men, after studying the design, said they would rely on a handyman in the village who would repair it or make a new one. All groups suggested a handle should be added to the flytrap so that it could be emptied without dirtying one's hands. It was pointed out by the men that the plastic sides of the trap would likely warp and eventually crack in direct sunlight during the hotter dry season.

## Discussion

We demonstrated a number of ways for reducing the production of *C. putoria* from latrines. An immediate reduction in fly numbers was seen with pyriproxyfen and soap, with a maximal reduction seen at 3- to 5-week post-treatment.

Pyriproxyfen has good residual activity in fresh water, with studies on mosquito control showing that it can be effective for 5–9 months after initial treatment (Yapabandara & Curtis 2002; Sihuincha *et al*. 2005). However, its persistence in latrines is unknown, although it is likely that the rich communities of bacteria in the latrine may mean that it is broken down more rapidly in dirty water than clean water. Clearly, further studies are needed to find the optimum dosage of pyriproxyfen for treating latrines and to determine how frequently latrines should be treated to suppress fly populations. One further possibility is that the use of pyriproxyfen dusts to treat latrines may result in the autodissemination of the active ingredient by adult flies dispersing into other latrines or faeces on the ground, resulting in more comprehensive control of *C. putoria* populations. Recent studies have shown that mosquitoes and houseflies (Devine *et al*. 2009; Geden & Devine 2012) can transfer pyriproxyfen from a dusted surface to a breeding habitat reducing the emergence of adult flies. On a cautionary note, tolerance to pyriproxyfen has been found in Diptera (Kawada *et al*. 1987; Bull & Meola 1994; Londershausen *et al*. 1996), so it is recommended that pyriproxyfen should not be used for extended periods or should be rotated or mixed with other insecticides to avoid the development of resistant flies.

Interestingly, adding soap to the latrines reduced fly numbers by 64%. Quite why this is so remains uncertain, but reducing the surface tension of the water surface may result in a proportion of the developing larvae drowning. Using latrine buildings for washing is common in rural Gambia, and the presence of soap may explain partly the highly variable production of flies from latrines, where about 20% of latrines produce 80% of flies (Lindsay *et al*. 2012). It is common practice in The Gambia for women who have finished their laundry to pour the waste water into the street. It would be relatively simple to get women to pour this water into latrines.

EPBs resulted in a 41% reduction in fly numbers. To work as a barrier to ovipositing and emerging adults, the beads must rest on the water surface, above the faeces. The treated latrines gave mixed results as in some latrines the height of the water column varied greatly from week to week. In some latrines, the beads were submerged after 2–3 weeks. Moreover, even where there was a surface covering of beads, the use of sticks and old clothes as a substitute for toilet paper would have provided a platform for faeces to accumulate on above the layer of beads.

We demonstrated that simple pit latrines can be turned into flytraps. We used odours generated from faeces contained in a latrine to attract flies to traps incorporated into the latrine design. Nonetheless, the overall numbers of flies collected in this manner were relatively small; we caught half the numbers of flies in our latrine traps compared with traps baited with fish nearby. There are a number of reasons for this discrepancy. Firstly, our latrines were newly constructed and were left for just 15 weeks before the start of our study. It is likely that a mature latrine may be more attractive to flies than a new one. Secondly, the vent pipe design is simple and airflow from the pipe was probably low, with the drop hole attracting more flies than the vent pipe itself. One solution to this is to make the vent pipe wider than the drop hole and place the vent pipe directly above the pit and the drop hole to one side. Further study of air circulation within the latrine is needed to find the best method for increasing airflow from the latrine into the trap.

The response of latrine users to the modified latrines was positive suggesting that such interventions would be well received by communities. The removal of flies and bad smells, as well as the absence of a burst of hot air that typically issues from a latrine after lifting the latrine lid, was appreciated by both men and women. Rather worryingly, children under 5 years of age were not allowed to use the latrines for fear of dirtying the area or the serious concern of falling into the latrine. In another study in The Gambia, 94% of 391 household heads stated that the youngest children were not allowed to use the latrine (Simms *et al*. 2005), confirming that this is a common practice in the country. Although it was reported from the FGDs that faeces from children were put into the latrine, this cannot be confirmed as no direct observations were made. However, in many instances observed by the investigators, this is not the case. Often the faeces of a young child are rolled in sand and then scooped onto an old plate with a flat object or broom before being placed on the compost. An earlier study in The Gambia reported that 46% of child faeces were thrown on the rubbish heap (Simms *et al*. 2005), and these may still attract flies. This is of concern for the control of diarrhoeal diseases. As we have demonstrated it is feasible to control fly production from latrines, it would be impracticable to control fly breeding on faeces, which are deposited on the ground, in and around the villages. Whilst the box trap is an efficient method for collecting *C. putoria* (Lindsay *et al*. in press), its durability in rural Gambia is unknown. Moreover, for the box trap to work over long periods, it would require regular removal and cleaning. Ideally, the trap should be at a height above ground that prevents interference from children, but still attracts flies. And it must be made from material that does not perish under strong sunlight. The design of a durable trap that is self-cleaning and therefore does not require the latrine owner to empty it would be an important goal of trap development.

## Conclusions

These preliminary findings demonstrate that it is possible to reduce the number of *C. putoria* produced from latrines. Pyriproxyfen was particularly successful although the duration of its effectiveness is not known. This study illustrates the proof of principle that latrines can be developed to serve as traps for latrine flies. These studies represent preliminary findings from our research and are published to encourage further work in this area as fly-free latrines are likely to contribute to a reduction in diarrhoeal diseases in communities in developing countries.

## References

[b1] Bull DL, Meola R (1994). Efficacy and toxicodynamics of pyriproxyfen after treatment of insecticide susceptible and -resistant strains of the house fly (Diptera: Muscidae). Journal of Economic Entomology.

[b2] Curtis C (2005). Insecticide-treated nets against malaria vectors and polystyrene beads against *Culex* larvae. Trends in Parasitology.

[b3] Curtis CF, Hawkins PM (1984). Entomological studies of onsite sanitation systems in Botswana and Tanzania. Transactions of the Royal Society of Tropical Medicine and Hygiene.

[b4] Curtis CF, Minjas J (1985). Expanded polystyrene for mosquito control. Parasitology Today.

[b5] Dawson S, Manderson L, Tallo VL (1993). A Manual for the Use of Focus Groups.

[b6] Devine GJ, Perea EZ, Killeen GF, Stancil JD, Clark SJ, Morrison AC (2009). Using adult mosquitoes to transfer insecticides to *Aedes aegypti* larval habitats. Proceedings of the National Academy of Sciences of the United States of America.

[b7] Dumpert J, Paterson K, Emerson P (2009). Performance assessment for the VIP toilet in the Upper West Region of Ghana. Waterlines.

[b8] Emerson PM, Simms VM, Makalo P, Bailey RL (2005). Household pit latrines as a potential source of the fly *Musca sorbens* - a one year longitudinal study from The Gambia. Tropical Medicine and International Health.

[b9] Geden CJ, Devine GJ (2012). Pyriproxyfen and house flies (Diptera: Muscidae): effects of direct exposure and autodissemination to larval habitats. Journal of Medical Entomology.

[b10] Hatakoshi M, Kawada H, Nishida S, Kisida H, Nakayama I (1987). Laboratory evaluation of 2 -(1-methyl-2-(4-phenoxyphenoxy) ethoxy) pyridine against larvae of mosquitoes and housefly. Japanese Journal of Sanitary Zoology.

[b11] Invest JF, Lucas J (2008). Pyriproxyfen as a mosquito larvicide.

[b12] Kawada H, Dohara K, Shinjo G (1987). Evaluation of larvicidal potency of insect growth regulator, 2-1-methyl-2-(4-phenoxyphenoxy)ethoxy pyridine, against the housefly, *Musca domestica*. Japanese Journal of Sanitary Zoology.

[b13] Laurence BR (1988). The tropical African latrine blowfly, *Chrysomya putoria* (Wiedemann). Medical and Veterinary Entomology.

[b14] Lindsay SW, Lindsay TC, Duprez J (2012). *Chrysomya putoria*, a putative vector of diarrheal diseases. PLoS Neglected Tropical Diseases.

[b15] Lindsay TC, Jawara M, d'Alessandro D, Pinder P, Lindsay SW Development of odour-baited flytraps for sampling the African latrine fly, *Chrysomya putoria*, a putative vector of enteric diseases. PLoS ONE.

[b16] Londershausen M, Alig B, Popsischil R, Turberg A (1996). Activity of novel juvenoids on arthropods of veterinary importance. Archives of Insect Biochemistry and Physiology.

[b17] Morgan PR (1977). The pit latrine-revived. Central African Journal of Medicine.

[b18] Muirhead Thomson R (1948). Studies on *Anopheles gambiae* and *A. melas* in and around Lagos. Bulletin of Entomological Research.

[b19] Reiter P (1985). A field trial of expanded polystyrene balls for the control of *Culex* mosquitoes breeding in pit latrines. Journal of the American Mosquito Control Association.

[b20] Rozendaal JA (1997). Vector Control: Methods for Use by Individuals and Communities.

[b21] Service M (1976). Mosquito Ecology. Field Sampling Methods.

[b22] Sihuincha M, Zamora-Perea E, Orellana-Rios W (2005). Potential use of pyriproxyfen for control of *Aedes aegypti* (Diptera: Culicidae) in Iquitos, Peru. Journal of Medical Entomology.

[b23] Simms VM, Makalo P, Bailey RL, Emerson PM (2005). Sustainability and acceptability of latrine provision in The Gambia. Transactions of the Royal Society and Tropical Medicine and Hygiene.

[b24] UNICEF/WHO (2012). Progress on drinking water and sanitation.

[b25] WHO (2006). Pesticides and their Application for The Control of Vectors and Pest of Public Health Importance.

[b26] Yapabandara AM, Curtis CF (2002). Laboratory and field comparisons of pyriproxyfen, polystyrene beads and other larvicidal methods against malaria vectors in Sri Lanka. Acta Tropica.

